# Debates in Allergy Medicine: Oral immunotherapy shortens the duration of milk and egg allergy - the con argument

**DOI:** 10.1186/s40413-018-0189-0

**Published:** 2018-06-15

**Authors:** Wenyin Loh, Mimi L. K. Tang

**Affiliations:** 1 Allergy and Immune Disorders, Murdoch Children’s Research Institute, Melbourne, Australia; 2 Allergy Service, Department of Paediatrics, KK Women’s and Children’s Hospital, Singapore, Singapore; 3 Department of Allergy and Immunology, The Royal Children’s Hospital, Melbourne, Australia; 4 Department of Paediatrics, University of Melbourne, Melbourne, Australia

**Keywords:** Food allergy, Oral immunotherapy, Milk, Egg, Desensitization, Sustained unresponsiveness, Tolerance

## Abstract

Oral immunotherapy (OIT) has been shown to be effective for inducing desensitization in children with cow’s milk and egg allergy. In contrast, there is limited evidence that OIT can induce tolerance or sustained unresponsiveness in food allergic patients. Sustained unresponsiveness, determined by a food challenge following a period of secondary avoidance, has been suggested to reflect a more enduring state of tolerance and is pertinent when considering the ability of OIT to shorten the duration of food allergy. While it has been shown that children who tolerate baked forms of egg and milk are more likely to develop tolerance compared to those who are allergic to baked forms of these foods, there is no convincing evidence that OIT using modified allergen in baked foods can hasten resolution of cow’s milk and egg allergy. Instead, it is likely that baked milk and baked egg tolerant children represent a sub-phenotype of milk and egg allergy that is more likely to resolve spontaneously over time.

## Background

Egg and cow’s milk are the commonest causes of food allergy in children. The prevalence of egg allergy is estimated to be 0.5–2.5% in western countries [1–3] and as high as 8.9% in Australia [4]. Similarly, prevalence rates of milk allergy range from 0 to 2% [1, 3]. Fortunately, most children develop natural tolerance with time - 50% of egg allergic children develop tolerance by 2–3 years of age [5, 6] and up to 80% by school-age [7]. Milk allergy also tends to resolve with about 50% of children developing tolerance by 4–5 years of age [8, 9]. Nevertheless, a significant number continue to have persistent egg and milk allergy as adults. Indeed, some studies suggest that rates of resolution may be reduced or delayed with disease persisting into adolescence in a larger proportion of children [10, 11].

## Oral immunotherapy outcomes: Desensitisation vs tolerance

Current oral immunotherapy (OIT) regimens typically involve the daily consumption of an allergen, commencing at a low dose followed by dose increments over several hours during the rush phase and periodically (usually every 2 weeks) during the build-up phase until the target maintenance dose is achieved. This maintenance dose is then continued on a daily basis for months to years or ongoing. Dose escalations during the rush and build-up phases are typically conducted under a physician’s supervision because of the risk of allergic reactions.

When considering the ability of OIT to shorten the duration of a food allergy, distinguishing between the outcomes of “desensitisation” and “tolerance” is important. Desensitisation is defined as an increase in threshold for reactivity that allows the patient to ingest increased amounts of a food without reaction while continuing on regular doses of that food (e.g., continuing OIT). This clinical unresponsiveness is temporary and is mediated by changes in effector cells (mast cells, basophils) without modulation of underlying pathogenic immune mechanisms; thus, the individual remains allergic to the allergen [12–15]. In contrast, tolerance is the ability to ingest unlimited amounts of a food without reaction even after discontinuation of the food indefinitely. It is presently not known whether OIT can induce true immune tolerance; hence, the term “sustained unresponsiveness” (SU) has been suggested [12] in place of “tolerance” when evaluating OIT efficacy to better differentiate a transient state of tolerance (disease remission) from a more permanent one (disease resolution). SU is believed to reflect sustained redirection of the immune response to allergen through the generation of regulatory T cells and/or allergen-specific anergy and clonal deletion [15, 16] and is expected to persist for at least months or years after immunotherapy has ceased.

Desensitisation can be determined clinically by performing a food challenge while a subject is still receiving OIT or eating regular doses of a food, and SU is confirmed by performing a food challenge after OIT or food intake has been stopped for a period of time. There is currently no consensus on the length of time that food/OIT intake should be stopped prior to challenge in order to demonstrate SU, but this is commonly in the range of 4–8 weeks [17]. Furthermore, the length of time that SU must persist to confirm true immunological tolerance remains unclear and it appears this initial state of “transient tolerance” may not be long-lived following OIT [18].

As all food allergies can resolve spontaneously over time, it is important to include a control or placebo treatment when evaluating the efficacy of a food allergy treatment. Therefore, in the next section we will focus primarily on randomised controlled trials (RCT) or meta-analyses of RCTs to address the question of whether OIT can shorten the duration of milk or egg allergy. Trials are further considered in relation to outcomes of desensitisation and SU, noting that duration of food allergy can only be shortened if there is attainment of SU (as against desensitisation) and moreover it is uncertain whether SU will be representative of true immune tolerance.

## OIT is effective for inducing desensitisation

OIT was first reported to successfully treat a child with egg anaphylaxis in 1908 [19]. Subsequent studies reported successful desensitisation in 57–94% of egg-allergic patients although studies were heterogeneous in design including differences in the target maintenance dose and duration of treatment [20–22]. A Cochrane review of OIT for egg allergy that considered trials published up to December 2013 included 4 randomised controlled trials with a total of 167 egg allergic children aged 4–15 years (100 OIT, 67 controls) [23]. One study used a placebo control while 3 used avoidance as the control. Successful desensitisation, defined as being able to tolerate a full serving of egg (10–13.6 g of egg protein or 10 mL raw egg white), was achieved in 39% of the OIT group compared to 11.9% of the control group, with a pooled relative risk ratio (RR) of 3.39 (95% confidence interval (CI) 1.74–6.62). Partial desensitisation (being able to tolerate 1–7.5 g of egg protein) was achieved in 79% of the OIT group compared to 13% of the control group, resulting in a pooled RR of 5.73 (95% CI 3.13–10.50). However, the range of treatment duration in the included studies was wide (6 to 22 months) and there was no adjustment for treatment duration. This is relevant because it has been suggested that a longer duration of treatment and higher maintenance dose may be associated with an increased likelihood of desensitisation, although this study did not include a parallel placebo-treated control group [24].

Similar rates of desensitisation are also noted for cow’s milk OIT [25, 26]. The first double-blind placebo-controlled trial (DBPCT) of cow’s milk OIT in 20 children showed a > 100-fold increase in threshold of milk protein tolerated (from 40 mg to 5140 mg of cow’s milk protein) [27]. A meta-analysis of five cow’s milk RCTs involving a total of 196 patients (106 OIT, 90 controls) found successful desensitisation in 62% of children in the OIT group compared to 8% of the control group (RR 6.61, 95%CI 3.51–12.44) [28].

Consistent with the understanding that underlying allergy persists in the desensitised state and reaction thresholds can fluctuate over time, long-term follow-up of children on home-based maintenance dosing has shown that a high proportion are unable to persist with regular allergen intake due to adverse reactions and severe reactions can occur among those who continue milk intake [29–32]. Keet et al., presented data on 32 children from 2 early cow’s milk OIT studies who were desensitised following OIT and followed-up for up to 5 years [29]. In both studies, participants were advised to continue regular cow’s milk intake following completion of the studies. After a median of 4.5 years in one study and 3.2 years in the second study, 16 (50%) of 32 participants were limiting their cow’s milk intake due to symptoms. Moreover, 22% (6/27) of participants who continued regular cow’s milk intake reported at least one episode of anaphylaxis in the preceding 12 months and one subject reported using intramuscular adrenaline at least twice a month for reactions to cow’s milk. Barbi et al., reported that among 132 patients desensitised to cow’s milk who continued on daily doses of cow’s milk, 64% experienced one or more reactions 2–84 months after hospital discharge with ~ 35% reporting 5 or more reactions [30]. In addition, 5 of 132 patients experienced serious reactions, requiring intramuscular adrenaline for “exacerbation of symptoms despite treatment, severe cyanosis, perception of a very severe crisis, loss of consciousness or collapse”. In another study by Paassilta et al., 16 of 28 (57%) participants were able to maintain regular cow’s milk intake up to 7 years after completion of treatment although 2 were limiting their intake because of cow’s milk-induced symptoms. While 19% of patients reported no milk-related symptoms at 7 years, 1 subject required intramuscular adrenaline for severe symptoms [32].

## What is the evidence that OIT is effective at inducing sustained unresponsiveness?

### OIT with whole egg and milk

Few studies have assessed for SU following egg or milk OIT and only 2 included a placebo group. Based on randomised trials, the ability for OIT to induce SU is uncertain [12, 22, 24, 33, 34]. An early RCT of egg and milk OIT [33] randomised 45 participants (median age 2.5 years, range 0.6–12.9 years) to receive OIT (11 egg OIT, 14 milk OIT) for 18–24 months or to continue avoidance (10 controls each for egg and milk). Sustained unresponsiveness was assessed by oral food challenge (OFC) performed after secondary elimination for 2 months; there was no difference in SU induction between participants receiving OIT (9 of 25; 36%) and those avoiding either egg or milk (7 of 20; 35%). In a DBPCT evaluating egg OIT, 55 children were randomised to receive OIT (*n* = 40) or placebo (*n* = 15) [11]. All subjects received double-blind placebo-controlled food challenges (DBPCFCs) to assess desensitisation at 10 months, following which placebo was stopped and children in the placebo group were followed through to 24 months while OIT was continued in the active group on an open-label basis. At 22 months, OIT was discontinued in the active group and DBPCFC was performed at 24 months (after 8 weeks elimination) to assess for SU. Subjects who received the placebo were only challenged if the egg-specific IgE was less than 2kU/L. Eleven of 40 (27.5%) egg allergic children who received 2 g per day of egg protein achieved SU compared with 0 of 15 placebo-treated children. However, this finding should be interpreted with caution as only 1 of 15 placebo participants had an egg-specific IgE level of less than 2kU/L and underwent the OFC to assess for SU at 24 months, whereas SU food challenges were completed for all OIT treated participants who had not withdrawn from the study which introduces a potential for bias. In a follow up study [23], OIT was continued in OIT treated participants who failed to achieve SU at 24 months until their egg sIgE fell below 2 kU/L. SU was achieved in 20 of 40 subjects (50%) after up to 4 years of treatment. However, this finding is difficult to interpret since there was no parallel placebo treated comparison group that was assessed for SU after a similar duration to control for natural resolution of egg allergy.

Two other studies in which participants received egg OIT for shorter periods but at higher doses have assessed for SU [22, 34]. Of 30 participants randomised to consume 1 egg every 48 h for 3 months, 37% achieved SU, as determined by DBPCFCs performed after 3 months of avoidance, compared to 1 of 31 (3%) children who continued avoiding egg [22]. Similarly, in a double-blind placebo-controlled trial, 5 of 16 (31%) subjects consuming 4 g per day of dehydrated egg white followed by 6 months ad libitum egg consumption achieved SU when assessed with DBPCFCs following 1 month of avoidance, compared to 1 of 14 (< 1%) placebo-treated children [34]. These findings suggest that duration of treatment and/or cumulative OIT dose (as a product of maintenance dose and the duration on maintenance dose) and not necessarily the maintenance dose reached may affect the likelihood of achieving SU.

The available evidence therefore indicates that although egg and milk OIT are effective at inducing desensitisation, the ability to induce SU or tolerance is limited using current protocols. It is possible that longer duration of treatment resulting in higher cumulative dose of allergen may lead to higher rates of allergy remission, and further studies are required to explore this.

### OIT using modified allergen

#### Inclusion of baked egg and milk in the diet

Food processing can affect the allergenicity of egg and milk proteins. For example, cooking at high temperatures can cause conformational changes in allergen epitopes, making these less allergenic. Both baked forms of egg and milk are less allergenic, not just due to alterations caused by heating but also from blocking access to epitopes through the formation of a food matrix with wheat [35, 36]. It has been shown that the majority of children with egg and milk allergy can tolerate baked forms of these foods [37, 38]. Furthermore, egg and milk allergic children who tolerate the allergen in its baked form are more likely to develop tolerance than those who react to the baked form [39]. In the HealthNuts study [5], egg allergic infants who were baked egg-tolerant were 5 times more likely to develop tolerance than those who were baked egg-allergic. Hence, it has been suggested that introduction of baked egg and milk into the diet using an OIT regimen may hasten resolution of these allergies; however, robust evidence for this remains lacking.

To assess the effect of baked egg ingestion on the natural history of egg allergy, Konstantinou et al., retrospectively evaluated 94 children who were either allergic (*n* = 55) or sensitised (*n* = 39) to egg [40]. Children were challenged with cake baked with 1 egg at study entry with 93% demonstrated to be tolerant. Tolerant subjects were instructed to continue daily consumption of baked egg with gradual increase in the egg content of the cake to a total of 1.5 g of egg protein and an open challenge to egg was performed at the end of 6 months. Of those consuming cake daily, 95% passed the open egg challenge leading the authors to conclude that consumption of baked egg (BE) might alter the natural course of the disease. However, a comparison group (BE-tolerant children who avoided BE) was not available to confirm this. Furthermore, clinical egg allergy was not confirmed in participants who were sensitised but had never ingested egg.

In another study, 79 egg allergic children underwent a BE challenge, of whom 56 were BE-tolerant and instructed to consume 1–3 servings of baked egg daily [41]. Subjects who ingested baked egg daily were offered open challenges to regular egg after 6 months if their egg sIgE was less than 2kU/L (or less then <7kU/L for children older than 7 years), while those who were BE-reactive were offered repeat open challenges to baked egg after 12 months. Those that were shown to be BE-tolerant at study entry were 12 times more likely to tolerate regular egg compared to those who were BE-reactive at study entry. Initially BE-tolerant subjects also developed regular egg tolerance earlier than initially BE-reactive subjects (41.7 months vs 57.5 months, *p* = 0.004). It was noted that once BE-reactive subjects become tolerant to baked egg, they were just as likely as initially BE-tolerant subjects to develop tolerance to regular egg. When compared to a retrospectively matched group of BE-allergic children who were strictly avoiding egg (*n* = 47), subjects in the active group developed tolerance to regular egg significantly earlier than those in the comparison group. The median time to regular egg tolerance was 50.0 months in the active group compared to 78.7 months in the comparison group (*p* < 0.0001). While these findings are encouraging, the lack of a control group (BE-tolerant subjects avoiding baked egg who undergo egg challenges at the same time points) makes it difficult to determine with confidence whether the intake of baked egg did indeed modify the natural history of disease. Furthermore, although the comparison group was matched for age, sex and sIgE, details of other characteristics that can influence persistence of egg allergy (eg. SPT wheal size, allergy to multiple foods) were not provided. Also, the decision to challenge the control patients to regular egg was based on individual allergist’s recommendations (and not at prespecified time points) so it is possible that some of these patients were already regular egg tolerant but not yet assessed as such.

Indeed, a recently published DBPC randomised trial involving 43 egg allergic children who were BE-tolerant randomised to consume 10 g of baked egg (1.3 g egg protein) 2–3 times per week for 6 months (*n* = 21) or similar egg-free baked goods (*n* = 22) found no between group difference in the development of tolerance to raw egg as assessed by OFC 1 month after ceasing study treatment - 4 of 17 (23%) children in the active group compared to 6 of 18 (33%) in the control group passed the raw egg challenge [42]. There was also no significant difference in egg sIgG4 levels between groups. This suggests that baked egg consumption does not alter the natural history of egg allergy in children already destined to be baked egg tolerant. The available evidence instead indicates that the ability to tolerate baked forms of egg identifies a subset or phenotype of egg allergic subjects who are more likely to outgrow their egg allergy and tend to do so earlier than those who are baked egg allergic; and furthermore, that resolution of egg allergy may progress along a continuum with tolerance to baked egg preceding tolerance to regular or raw egg.

Kim et al. evaluated 88 milk allergic children who, based on an initial baked milk challenge, were classified as BM-tolerant or BM-reactive [43]. Those who were tolerant were instructed to incorporate baked milk products into their diets following which unheated milk challenges were performed after 6 months. A comparison group was retrospectively gathered. BM-tolerant subjects were 28 times more likely to tolerate unheated milk compared to those who were BM-reactive and subjects regularly consuming baked milk were 16 times more likely to tolerate unheated milk compared with the comparison group. Similar findings were noted in other studies [44, 45]. Nevertheless, once again, lack of suitable control groups (BM-tolerant subjects who avoid baked milk with equivalent outcome assessments) in these studies limit the interpretation of these findings.

#### OIT with baked egg or milk

Several studies have evaluated the ability of OIT with modified allergen to allow subjects to tolerate unmodified whole allergen. Bravin et al., explored the safety and efficacy of baked egg (BE) OIT in allowing subjects to ingest whole egg without reaction. Fifteen BE-allergic children were instructed to eat biscuits containing egg protein increased daily over 60 days to a maximum dose of 6.25 g [46]. Those who achieved the maximum dose then underwent an OFC with boiled egg. Eight subjects completed the OIT protocol and all of these subjects passed the OFC; hence, 53% of children who received BE OIT were able to tolerate a whole boiled egg at the end of the OIT protocol. The remaining 7 children did not complete the OIT protocol - 2 were unable to proceed beyond the first dose because of allergic symptoms and 5 achieved partial desensitisation to whole egg allowing them to consume trace amounts of egg.

Goldberg et al. evaluated the efficacy of baked milk (BM) OIT in enabling BM-allergic patients who had failed milk OIT previously to tolerate whole cow’s milk [47]. Fifteen patients who had reacted to 30 mg or less of unheated milk protein during a previous OIT program were given daily doses of BM that was increased monthly to a maximum of 1.3 g per day over 12 months. Cow’s milk OFCs were performed after 6 and 12 months of BM treatment. Only 3 subjects tolerated 1.3 g per day of BM (20% full desensitisation), and 8 did not complete the program because of IgE-mediated reactions. More importantly, it was noted that patients frequently developed reactions to doses they were previously tolerating for more than a month, including 1 patient who achieved maintenance dose only to later regress because of continued reactions.

In a recent study evaluating the effect of more frequent versus less frequent introduction of more allergenic forms of milk (MAFM) on progression to SU, milk allergic children who were BM tolerant were randomised to undergo 6 monthly versus 12 monthly escalations of progressively less heat-denatured forms of milk (muffin < pizza < rice pudding < non-baked liquid milk) over 36 months [48]. Subjects who eventually tolerated non-baked liquid milk for 3 months were then placed on strict CM avoidance for 1 month after which an OFC was performed. Of 136 subjects enrolled, 41 (30%) were BM reactive while 85 (63%) were BM tolerant at baseline. Overall, 41 of 85 (48%) BM-tolerant children compared to 0 of 41 BM-reactive children tolerated non-baked liquid milk at the 36 month OFC with no difference noted between the 6- and 12-month escalation groups. Of the 22 children who went on to discontinue milk intake for 1 month, all passed the final milk OFC and successfully introduced milk into their diets.

In all of these studies, the absence of a control group (BE- or BM-tolerant subjects who continued to avoid baked egg or baked milk) makes it difficult to determine whether the OIT regimens increased acquisition of SU.

#### OIT with hydrolysed egg

In the only randomised placebo-controlled study of modified allergen OIT, Giavi et al., [49] showed that OIT using hydrolysed egg was not effective at inducing the ability to ingest whole egg without reaction. Twenty-nine egg-allergic children were randomised to receive daily doses of a low allergenic hydrolysed egg product (*n* = 15) or placebo (*n* = 14) for 6 months. Eleven actively treated subjects completed the protocol. Four of the 15 (26.7%) subjects who received HydE OIT passed the OFC compared to 3 of 14 (21%) subjects in the placebo group (p = NS). Furthermore, while all subjects in the HydE group tolerated the full maintenance dose at the first visit, all except 1 experienced at least 1 adverse event during the course of the treatment. However there were no serious adverse events and adrenaline was not required.

## Is tolerance achievable with OIT?

In order to hasten resolution of food allergy, it would be necessary to induce a persistent state of tolerance and, at this time, it remains unknown whether the attainment of sustained unresponsiveness is equivalent to acquisition of true immune tolerance. There is only one study that has assessed for persistence of SU following OIT [18]. Syed et al. followed 20 patients with peanut allergy who completed 24 months of peanut OIT [18]. Of the 7 subjects who achieved SU at 3 months post-peanut OFC, 3 lost their SU status by 6 months post-treatment, suggesting that OIT-induced SU is in some cases short-lived. Given that this was a small cohort study performed at a single site, larger trials are needed to clarify whether OIT-induced SU can indeed be long-lived, at least in a subset of individuals.

## Conclusion

Currently, there is no convincing evidence that OIT using either whole protein or modified allergen in baked foods can influence the natural history of egg or milk allergy. Findings from limited randomised and open controlled trials suggest that OIT can induce SU in only a small subset of participants. Furthermore, while it has been shown that children who tolerate baked forms of egg and milk are more likely to develop tolerance than those who are unable to tolerate baked forms of these foods, there is insufficient evidence that ingestion of baked food is important in hastening tolerance development. 

It is equally (if not more) plausible that the ability to tolerate baked forms of egg or milk identifies a sub-phenotype of egg and milk allergy that is transient, and/or the natural course for resolution of egg and milk allergy involves the sequential acquisition of tolerance firstly to baked forms of egg or milk followed by tolerance to unheated native forms of egg and milk.

## Abbreviations

BE: Baked egg
BM: Baked milk
DBPCFC: Double-blind placebo-controlled food challenge
DBPCT: Double-blind placebo-controlled trial
OFC: Oral food challenge
OIT: Oral immunotherapy
RCT: Randomised controlled trial
SU: Sustained unresponsiveness
